# Influence of Extraction Solvent on the Phenolic Profile and Bioactivity of Two *Achillea* Species

**DOI:** 10.3390/molecules26061601

**Published:** 2021-03-13

**Authors:** Dominika Kaczorová, Erna Karalija, Sabina Dahija, Renata Bešta-Gajević, Adisa Parić, Sanja Ćavar Zeljković

**Affiliations:** 1 Centre of the Region Haná for Biotechnological and Agricultural Research, Department of Genetic Resources for Vegetables, Medicinal and Special Plants, Crop Research Institute, Šlechtitelů 29, 78371 Olomouc, Czech Republic; dominika.kaczorova@vurv.cz; 2 Centre of Region Haná for Biotechnological and Agricultural Research, Palacky University, Šlechtitelů 27, 78371 Olomouc, Czech Republic; 3 Laboratory for Plant Physiology, Department of Biology, Faculty of Science, University of Sarajevo, Zmaja od Bosne 33-35, 71 000 Sarajevo, Bosnia and Herzegovina; erna.karalija@gmail.com (E.K.); sabina_dudevic@yahoo.com (S.D.); adisacausevic@hotmail.com (A.P.); 4 Laboratory for Microbiology, Department of Biology, Faculty of Science, University of Sarajevo, Zmaja od Bosne 33-35, 71 000 Sarajevo, Bosnia and Herzegovina; renata_besta@yahoo.ca

**Keywords:** *Achillea lingulata* Waldst, *Achillea abrotanoides* Vis, extraction, phenolic compounds, antioxidant activity, antimicrobial activity

## Abstract

The phenolic composition, as well as the antioxidant and antimicrobial activities of two poorly investigated *Achillea* species, *Achillea lingulata* Waldst. and the endemic *Achillea abrotanoides* Vis., were studied. To obtain a more detailed phytochemical profile, four solvents with different polarities were used for the preparation of the plant extracts whose phenolic composition was analyzed using UHPLC-MS/MS (ultra-high performance liquid chromatography-tandem mass spectrometry). The results indicate that both of the investigated *Achillea* species are very rich in both phenolic acids and flavonoids, but that their profiles differ significantly. Chloroform extracts from both species had the highest yields and were the most chemically versatile. The majority of the examined extracts showed antimicrobial activity, while ethanolic extracts from both species were potent against all tested microorganisms. Furthermore, the antioxidant activity of the extracts was evaluated. It was found that the ethanolic extracts possessed the strongest antioxidant activities, although these extracts did not contain the highest amounts of detected phenolic compounds. In addition, several representatives of phenolic compounds were also assayed for these biological activities. Results suggest that ethanol is a sufficient solvent for the isolation of biologically active compounds from both *Achillea* species. Moreover, it was shown that the flavonoids naringenin and morin are mainly responsible for these antimicrobial activities, while caffeic, salicylic, chlorogenic, *p*-coumaric, *p*-hydroxybenzoic, and rosmarinic acid are responsible for the antioxidant activities of the *Achillea* extracts.

## 1. Introduction

Selection of an appropriate solvent and extraction protocol is the key for successful isolation of biologically active compounds from medicinal plants. The extraction solvents are chosen according to their polarity, and therefore, the ability to isolate specific types of compounds with different structures and physicochemical properties. The solvents accepted for use in pharmaceutical formulations are water, ethanol, and glycerol [1]. The polarity of extraction solvents influences the extraction efficiency of phenolic compounds. Less polar solvents extract smaller amounts of phenolic compounds, and therefore, these extracts possess a lesser potential for scavenging free radicals [2]. Generally, highly hydroxylated aglycone forms of phenolic compounds are soluble in water, alcohols (ethanol, methanol), and their mixtures, while less polar and highly methoxylated aglycone forms are extracted into less polar solvents (ethyl acetate, acetone, chloroform), [3,4]. Since hydroxy groups of phenolic compounds contribute to antioxidant activity, more polar extracts generally possess higher antioxidant activities.

Even though the use of non-toxic solvents is more desirable, some phytochemicals with hydrophobic properties are necessary for extraction by non-polar solvents. Artemisinin could be mentioned as an example. It is a highly active antimalarial compound isolated from *Artemisia annua* that is extracted from the plant material using non-polar solvents, such as petrol ether and hexane. However, the use of hydrocarbon solvents is not environmentally friendly, and even after evaporation, the solvents could be still unintentionally present in the sample in trace amounts. However, the extraction of artemisinin with water is inefficient and ethanol extraction causes rapid degradation of the compound [5]. Another option for the extraction of non-polar analytes could be supercritical fluid extraction (SFE). The significant benefit of this type of extraction is the non-use of flammable and toxic solvents, which efficiently extract phenolic compounds [6], but also other bioactive molecules of interest [7] from different plant materials. The efficiency of SFE was tested on *Achillea millefolium* and resulted in an almost threefold increase in the concentration of total phenolic compounds in comparison with ethanolic extraction [8]. Ultrasound-assisted extraction is widely used for extracting compounds from plant material. This technique is based on the disruption of plant cells and the liberation of the compounds to the solvent under low temperatures, preventing the degradation of thermolabile natural metabolites [9]. It is very convenient for the isolation of phenolic compounds. Thus, these extracts may also have better antioxidant activity in comparison to the extracts from Soxhlet extraction and maceration [10]. Moreover, the use of ultrasound-assisted extraction reduces energy costs and extraction times [11].

The human population encounters different pathogens, including urinary tract infection pathogens. These infectious microorganisms are mainly *Escherichia coli* [12], *Salmonella* sp. [13], and *Staphylococcus aureus*, a foodborne [14], skin [15], and soft tissue infection pathogen [16]. Due to the extensive use of antibiotics, both *E. coli* [17], and *S. aureus* [18] are common pathogens that have developed multiple drug resistances. However, some antibiotics show higher activities when combined with medicinal plant extracts [19]. A rising number of pharmaceutical companies develop herbal remedies to be used as a replacement for or a supplement to conventional medicines [20], primarily as prevention against disorders. Some examples are members of the genus *Carpobrotus* [21] or *Hedychium* [22]. Another interest is the development of safer antioxidants from natural sources to substitute for synthetic antioxidants (BHT, BHA) with potential health risks [23].

Plants of the Asteraceae family have been widely used as traditional medicinal herbs since ancient times. They are the source of many compounds that possess antioxidant, antimicrobial, and anticancer properties [24]. Moreover, extracts of Asteraceae plants showed high efficiency in the treatment of diabetes, inflammations, etc. [25], and also in cardiovascular-related diseases [26]. This highly diverse family is mainly distributed in Europe and the northern hemisphere. In general, plants of the genus *Achillea* contain a broad spectrum of compounds with bioactive properties. They are reported as tonic, anti-inflammatory, anti-spasmodic, diaphoretic, diuretic, and emmenagogic agents and have been used for the treatment of hemorrhages, pneumonia, rheumatic pain, and wound healing [27]. Their protective and antioxidant activities are linked with their content of phenolics and flavonoids [28]. Furthermore, the chemistry of the *Achillea* species could be used for the chemotaxonomical description of this genus [29,30]. Moreover, *Achillea millefolium* L. is listed as a plant drug in the Czech Pharmacopoeia [31].

**Figure 1:**
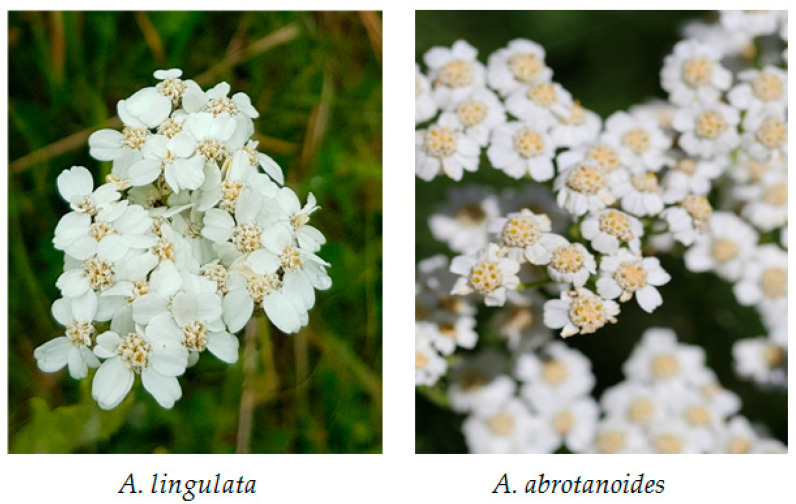
Inflorescences of the investigated *Achillea* species.

The present work is comprised of the identification and quantification of the compounds from the vegetative parts and inflorescences of *Achillea lingulata* Waldst. and *A. abrotanoides* Vis. (Figure 1). *Achillea lingulata* grows in the southeastern parts of Europe, with the main distribution in the Balkan Peninsula, Carpathians, and Belarus, while *A. abrotanoides* is endemic to the Balkan Peninsula. To isolate diverse compounds from these plants, four solvents with different polarities were used and all extracts were characterized by their phenolic composition and antioxidant and antimicrobial activities. To the best of our knowledge, there is little data about the phenolic composition and bioactive compounds in the roots [32] and aerial parts [33] of *A. lingulata*, and no data for *A. abrotanoides*.

## 2. Results and Discussion

### 2.1. Extraction

The objective of the extraction process is to maximize the amount of target compounds and to obtain the highest biological activity of these extracts. The extraction yield and biological activity of the resulting extract were affected by both the extraction technique and the extraction solvent. Ultrasound-assisted extraction, or sonication, uses cavitation energy in the solvent that accelerates the dissolution and diffusion of the solute as well as the heat transfer, which improves the extraction efficiency. This extraction technique requires low solvent and energy consumption and allows for the reduction of extraction temperature and extraction time. Therefore, it is applicable for the extraction of thermolabile and unstable compounds [34].

Several factors should be considered in the selection of solvents, mainly selectivity, solubility, cost, and safety [35]. In general, alcohols, acetone, and water are used for the extraction of bioactive compounds from the plant material, but the selection is based on the properties of the compound of interest, as well as the plant material used [34].

Plant extracts were prepared through the sonication of dry plant material (vegetative part and inflorescence) in petrol ether, chloroform, ethanol, and water. The yields of the extracts are presented in Table 1, from which it can be seen that the chloroform extracts had the highest yields for both plant species and their investigated organs. Usually, this halogenated solvent with medium polarity can isolate a wide range of compounds from plant material and is commonly used for the non-targeted isolation and analysis of natural products [23]. However, due to its toxicity in long exposures, chloroform is not a suitable solvent for use in pharmaceutical preparations. Furthermore, water extracts also showed relatively high yields for both plants and their organs. On the contrary, petrol ether and ethanol revealed the lowest yields.

### 2.2. UHPLC-MS/MS Analysis of Phenolic Compounds

A UHPLC-MS/MS (ultra-high performance liquid chromatography-tandem mass spectrometry) analysis was used for the separation and identification of phenolic compounds in the extracts of these two *Achillea* species. This is the first report of the phenolic composition of aerial parts of both *A. lingulata* and *A. abrotanoides*. To the best of our knowledge, only reports on the composition of the roots [32], lignans [33,36], and volatile compounds [37,38,39,40] for *A. lingulata* are recorded.

**Figure 2:**
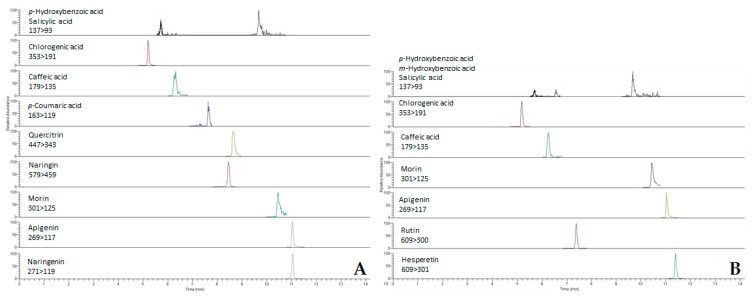
MRM chromatograms of ethanolic extracts of inflorescences of *A. abrotanoides* (**A**) and *A. lingulata* (**B**).

Among the 32 phenolic compounds analyzed, 21 phenolic acids and flavonoids were detected and quantified in the extracts of these two *Achillea* species (Table 2). In general, *A. abrotanoides* was found to be richer in phenolic compounds than *A. lingulata*. As an example, the (multiple reaction monitoring (MRM) chromatograms of ethanolic extracts of inflorescences of both investigated *Achillea* species are presented in Figure 2.

**Figure 3:**
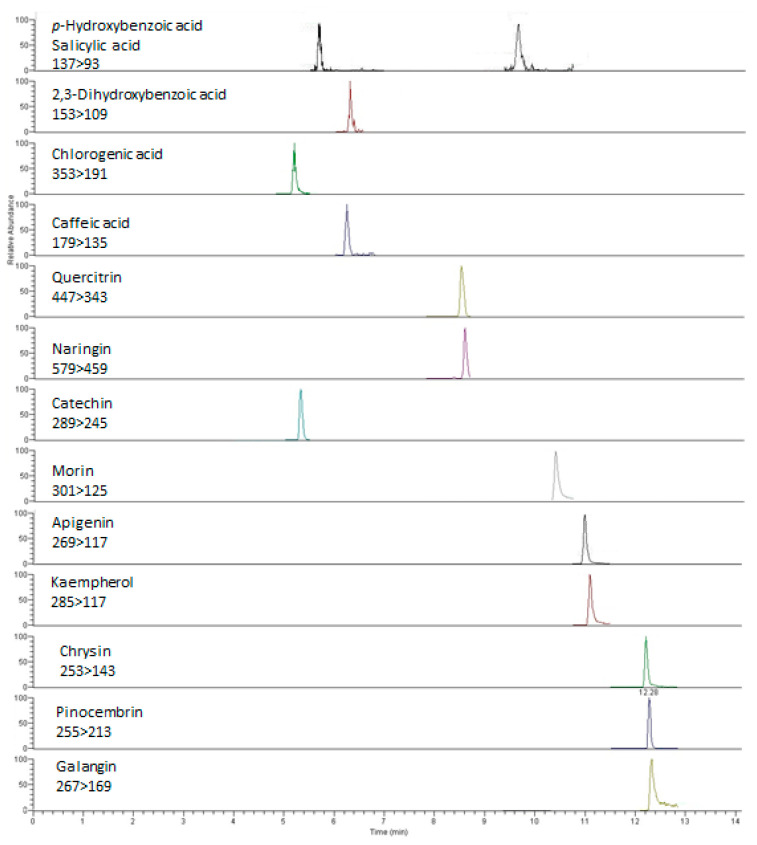
MRM chromatograms of the chloroform extract of vegetative parts of *A. abrotanoides*.

Chloroform as a solvent revealed the greatest diversity in phenolic profiles of both investigated plants. There were 7 phenolic acids and 9 flavonoids found in the vegetative part of *A. abrotanoides*, while 12 phenolic compounds in total were found in the extracts of *A. lingulata*. The highest concentration of phenolic compounds was detected in the chloroform extract of the inflorescence of *A. abrotanoides* (915.3 µmol/g), for which the MRM chromatograms are presented in Figure 3. As expected, the lowest content of phenolics was found in petrol ether extract of the vegetative part of *A. lingulata* (total concentration 2.1 µmol/g).

Among hydroxybenzoic acids investigated, *p*-hydroxybenzoic acid was the most abundant in the extracts of *A. abrotanoides* (up to 205.073 ± 8.626 µmol/g), while the levels of salicylic acid were quite similar in all examined extracts of both species. Other hydroxybenzoic acids are mostly found in polar extracts, i.e., ethanolic and aqueous. In addition, the levels of chlorogenic and caffeic acids, cinnamic acids commonly found in plants, were relatively low in comparison with the levels of hydroxybenzoic acids (Table 2). On the contrary, significant levels of rosmarinic acid were found in the inflorescence of the endemic *A. abrotanoides* (up to 207.473 ± 17.557 µmol/g). Unexpectedly, *p*-coumaric acid was not a dominant phenolic acid in *A. abrotanoides*, and it was not detected in *A. lingulata*. However, this hydroxycinnamic acid was identified in many other yarrows, such as *A. millefolium*, *A. distans*, *A. biserratae*, and *A. beibrestinii* [41,42,43,44].

Furthermore, apigenin was found to be the most abundant flavonoid found in the vegetative parts of both *Achillea* species, with levels of up to 112.010 ± 6.564 µmol/g and 165.688 ± 9.680 µmol/g for *A. abrotanoides* and *A. lingulata*, respectively. Apigenin was suggested as the main flavonoid in other *Achillea* species, such as *A. distans*, *A. ligustica*, *A. collina*, *A. millefolium*, etc. [41,44,45,46,47]. Moreover, isoquercitrin and rutin were the main flavonoids in *A. schurii* in the study of Benedec et al. [48]. On the contrary, the inflorescences of the investigated *Achillea* species differ in their flavonoid profiles, i.e., *A. abrotanoides* was rich in naringenin (up to 362.662 ± 4.922 µmol/g), while the flowers of *A. lingulata* contained notable amounts of hesperetin and rutin (Table 2).

### 2.3. Antimicrobial Activity

All isolated extracts, together with the representatives of the phenolic compounds detected, were tested for antimicrobial activity using the diffusion method. The results are summarized in Table 3. This is the first report of antimicrobial activity for the aerial plant parts of *A. abrotanoides*, while the antimicrobial activity of *A. lingulata* aerial parts has been studied before [49]. However, this is the first attempt to compare different extraction solvents that isolate the phenolic compounds that might be responsible for the antimicrobial activities of *A. lingulata* and *A. abrotanoides*.

All four water extracts of both *Achillea* species showed no antimicrobial potential, whereas they contained significant amounts of phenolic compounds (Table 2). This result was also reported previously when aqueous extracts exhibited no antimicrobial activity [50], which might be attributed to the inefficient diffusability of aqueous solutions in the agar medium [51], as well as to the lesser ability of aqueous extracts to damage microbe cell walls [52]. However, ethanol extracts of both species showed strong antimicrobial effects against all tested microorganisms except for *Staphylococcus aureus* with an equal or even higher efficiency than the antibiotic ampicillin or the antimycotic nystatin that were used as positive controls (Table 3). In general, all inflorescence extracts of both species showed more potent antimicrobial activities than the same extracts from the vegetative parts. The inflorescence ethanolic extract of *A. abrotanoides* was significantly more effective against *Enterococcus faecalis* compared to the ampicillin. All extracts (except for the chloroform extract of the vegetative part of *A. abrotanoides*) exhibited the same or even more potent activity against *Candida albicans* than the antimycotic. In this case, the chloroform extract of *A. abrotanoides* inflorescence had two times greater an antimicrobial effect than nystatin. Moreover, *E. coli* was equally as susceptible to nearly all extracts as to the reference antibiotic.

According to the available literature, other *Achillea* species did not demonstrate such potent antifungal activity against *Candida albicans*. The inhibition zone of ethanol flower extracts of *A. schurri* was recorded to be only 6 mm [49], while significantly higher values were recorded for the *A. lingulata* ethanol extract and the *A. abrotanoides* chloroform extract (Table 3). A similar observation was recorded for *A. millefolium* by Maz et al. [53]. No activity of certain *A. millefolium* plants growing at an average altitude against *E. coli* was recorded and the activity was limited to Gram-positive bacteria, while the *A. abrotanoides* and *A. lingulata* extracts were as effective as the reference antibiotic.

In addition, several representatives of each class of phenolic compounds at a concentration of 0.1 mg/mL were also assayed for their antimicrobial activities (Table 3). Among them all, the flavonoid morin was the only compound that successfully inhibited the growth of all four microorganisms. This compound was found in the ethanolic extract of the inflorescences and vegetative parts of both investigated *Achillea* species. Moreover, the flavonon naringenin showed very high activity against *E. faecalis*, *S. aureus*, and *C. albicans*, with the inhibition zones being bigger than those of ampicillin and nystatin. High concentrations of naringenin were found in the chloroform and ethanolic extracts of *A. abrotanoides*. Therefore, the activity of these extracts might be explained by the presence of this phenolic compound. Representatives of both hydroxybenzoic and hydroxycinnamic acids revealed similar activities, lower than the activities of flavonoids (Table 3), which is in agreement with the literature data [54].

Antimicrobial activity of phenolic compounds might be explained by the modification of the permeability of cell membranes, the changes in various intracellular functions induced by the hydrogen binding of the phenolic compounds to enzymes, or by the modification of the cell wall rigidity [55,56,57]. Phenolic acids have been shown to disrupt membrane integrity, as they cause consequent leakage of essential intracellular constituents [58], while flavonoids may link to soluble proteins located outside the cells and with bacteria cell walls, thus promoting the formation of complexes [57,59].

### 2.4. Antioxidant Activity

**Figure 4:**
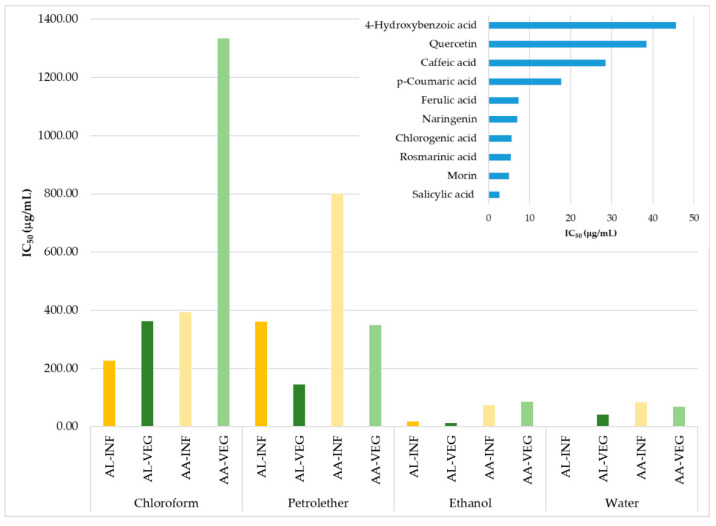
Antioxidant activity of the extracts of two *Achillea* species and their selected phenolic compounds. AA = *Achillea abrotanoides*, AL = *Achillea lingulata*, INF = inflorescence, VEG = vegetative part.

Antioxidant activity of the aerial plant parts of *A. lingulata* and *A. abrotanoides* has not been described previously. In this study, the DPPH (2,2-diphenyl-1-picrylhydrazyl) radical scavenging activity of all the extracts was tested and expressed as an IC_50_ value, which represents the concentration of the extracts that scavenge 50% of the radicals. In addition, selected phenolic compounds were also assayed for their abilities to scavenge stable DPPH radicals. All results are presented in Figure 4. In general, all extracts of *A. lingulata* showed a stronger antioxidant potential compared to *A. abrotanoides*.

The polar aqueous and ethanolic extracts of both investigated species had the lowest IC_50_ values. The most potent extract was the water inflorescence extract of *Achillea lingulata*, with an IC_50_ value of 1.52 µg/mL. This extract contained significant levels of rosmarinic acid, which also possesses strong antioxidant activity against stable DPPH radicals (IC_50_ 5.47 µg/mL). Moreover, the activity of this extract could be attributed to other phenolic compounds that are not quantified due to the lack of standards. Ethanol extracts of the inflorescence, as well as the vegetative part of *Achillea lingulata*, also showed a high antioxidant capacity, with IC_50_ values of 18.14 and 12.73 µg/mL, respectively. These extracts were rich in *p*-hydroxybenzoic acid, but also the flavonoid apigenin. The antioxidant potential is decreased via a decrease in the polarity solvent used [60,61], and the chloroform extracts of *A. abrotanoides* had the lowest antioxidant potential and the highest IC_50_ values (Figure 4). On the contrary, chloroform extracts of the vegetative parts and the inflorescence were rich in the phenolic compounds detected. Therefore, its low ability to scavenge stable radicals could be explained by the fact that chloroform isolates a high amount of chlorophylls and other pigments from leaves. These compounds do not possess significant antiradical activities [62].

The antioxidant activity of the extracts was correlated with the concentrations of the selected phenolic compounds, and it was found that caffeic (R = 0.6256), salicylic (R = 0.6296), chlorogenic (R = 0.6394), *p*-coumaric (R = 0.6402), *p*-hydroxybenzoic (R = 0.7493), and rosmarinic acid (R = 0.9807) have very high positive correlations with the antioxidant activity. Therefore, it might be concluded that these acids are responsible for the antioxidant activity of the extracts that contain them.

DPPH radical scavenging potential has been previously recorded for other *Achillea* species [48,63], ranging from the low IC_50_ of 0.52 µg/mL, as recorded for the essential oils of *A. pannonica* [64], up to 1.172 µg/mL for extracts of *A. eriophora* [65]. For some extracts, such as water and ethanol extracts of *A. lingulata* and *A. abrotanoides*, reported IC_50_ values are even lower than those of some synthetic or natural antioxidants [50,66,67,68].

## 3. Materials and Methods

### 3.1. Plant Material

Samples of *A. lingulata* and *A. abratonoides* were collected during the flowering stage at Mt. Jahorina (June 2017) and Mt. Bjelašnica (July 2017), respectively (Figure 1). Specimens were deposited at the herbarium of the Department of Biology, Faculty of Science, the University of Sarajevo under the voucher no. LERP 355 and LERP 356. The inflorescence and the vegetative parts were separated and air-dried for 7 days at room temperature (23 °C) in a shaded, well-ventilated laboratory. Dried samples were finely powdered in the mill and stored at 4 °C until use.

### 3.2. Chemicals

Standards of phenolic acids and flavonoids (apigenin, 2,3-dihydroxybenzoic acid, caffeic acid, catechin, chlorogenic acid, chrysin, ferulic acid, galangin, gallic acid, hesperidin, *m*-hydroxybenzoic acid, *p*-hydroxybenzoic acid, 5-hydroxyferulic acid, kaempferol, methyl *p*-coumarate, morin, myricetin, naringenin, naringin, *p*-coumaric acid, pinocembrin, quercetin, quercitrin, rosmarinic acid, rutin, salicylic acid, salicylic acid glucoside, sinapic acid, syringic acid, trans-cinnamic acid, vanillic acid, *p*-coumaric acid-d_6_, and salicylic acid-d_4_) were purchased from Sigma-Aldrich (Steinheim, Germany).

The sodium molybdate dihydrate, sodium nitrite, sodium hydroxide, sodium carbonate, hydrogen peroxide, sodium ascorbate, dimethyl sulfoxide (DMSO), and bovine hemoglobin were also purchased from Sigma-Aldrich (Steinheim, Germany). The LC (liquid chromatography) grade methanol, analytical grade orthophosphoric acid, hydrochloric acid, aluminum chloride, sodium acetate, ethanol, and Folin–Ciocalteu reagent were purchased from Merck (Darmstadt, Germany). The DPPH (2,2-diphenyl-1-picrylhydrazyl) was obtained from Alfa-Aesar (Karlsruhe, Germany). All spectrophotometric data were acquired using a Jasco V-530 UV-Vis spectrophotometer (Jasco International Co., Ltd., Tokyo, Japan).

### 3.3. Preparation of the Extracts

For the inflorescence and vegetative aerial parts, 500 mg each of the powdered plant material was soaked in 12.5 mL of solvent (petrol ether, chloroform, ethanol, and water) and sonicated for 30 min at 23 °C. The supernatant was transferred into a new vial and the sediment was soaked again in the same solvent. Due to the high evaporation rate of petrol ether and chloroform, these extracts were evaporated to dryness and resuspended in DMSO.

### 3.4. UHPLC-MS/MS Analysis of the Extracts

Standard solutions of the target phenolic compounds were first prepared in methanol at 1 mM concentrations, and the solutions were gradually diluted in the mobile phase to the working concentrations that ranged from 0.01 to 50 µM. Quantification was performed by the isotope diluting method using *p*-coumaric acid-*d*_6_ and salicylic acid-*d*_4_.

UHPLC-MS/MS was performed on an UltiMate™ 3000 liquid chromatographic system consisting of binary pumps, an autosampler, and a column thermostat coupled to a TSQ Quantum Access Max triple quadrupole mass spectrometer (Thermo Fisher Scientific, Waltham, MA, USA). Chromatographic separation was performed on an Acquity BEHC18 (150 × 3.0 mm; 1.7 µm particle size) UHPLC column (Waters Corp., Milford, MA, USA) kept at 40 °C. The mobile phase consisted of 10 mM formic acid in water (component A) and acetonitrile (component B). Compounds were separated using a binary gradient starting at 5% B for 0.8 min, increasing to 10% B for 0.4 min with an isocratic run for 0.7 min, then at 15% B for 0.5 min with an isocratic run for 1.3 min, at 20% B for 0.3 min with an isocratic run for 1.2 min, at 25% B for 0.5 min, at 35% B for 2.3 min, at 70% B for 2.5 min, then at 100% B for 1 min with an isocratic run for 1 min, and then back to 5% B for 0.5 min. Finally, the equilibration to initial conditions took 3.3 min, with a total chromatographic run of 16 min. The flow rate was 0.4 mL/min and the injection volume was 10 µL.

All analytes were detected in negative electrospray ionization mode (ESI-). Multiple reaction monitoring (MRM) mode was used for their quantification. The spray voltage was 3 kV, the temperature of the ion transfer tube vaporizer was 320 °C, the sheath gas pressure was 45 psi, and the auxiliary gas pressure was 15 psi.

### 3.5. Determination of Antioxidant Activity

DPPH antioxidant activity was evaluated for all extracts and standards according to Meda et al. [69]. Briefly, 200 µL of sample solution was mixed with 50 µM of DPPH solution in ethanol, and the absorbance of the tested mixtures was measured at 517 nm after 30 min. Absolute ethanol was used to zero the spectrophotometer, DPPH solution was used as a blank sample, and different phenolic compounds (Figure 4) were used as a positive probe. Antioxidant activity was calculated as IC_50_, which is referred to as the concentration of extract that scavenges 50% of free DPPH radicals.

The radical-scavenging activities of the tested samples, expressed as a percentage of the inhibition of DPPH, were calculated according to the formula IC (%) = [(A_0_ − A_t_)/A_0_] % 100, where A_0_ and A_t_ are the absorbance values of the blank sample and the test sample at 0 and 30 min, respectively. Four different concentrations of each sample were assayed. Percent inhibition after 30 min was plotted against concentration, and the equation for the line was used to obtain the IC_50_ value. A lower IC_50_ value indicates greater antioxidant activity.

### 3.6. Determination of Antimicrobial Activity

Agar well diffusion method was used to evaluate the antimicrobial activity of the plant extracts and standards according to National Committee for Clinical Laboratory Standards (NCCLS) [70,71]. Each well contained 100 µL of extract or standard. Bacterial strains used in the analysis included the Gram-positive bacteria *E. faecalis* ATCC^®^ 19433TM and *S. aureus* subsp. *aureus* ATCC^®^ 6538TM and the Gram-negative bacteria *S. abony* NCTC^®^ 6017TM, *E. coli* ATCC^®^ 8739TM, and the yeast *C. albicans* ATCC^®^ 10231TM. Bacterial strains were used as a standardized inoculum of 5×10^5^ CFU/mL using a McFarland standard [72].

Müller–Hinton and Sabouraud medium were used for the cultivation of the bacterial strains and yeast, respectively. Ampicillin was used as a positive standard for bacterial strains and nystatin for *Candida albicans*. Ethanol and DMSO were used as negative controls. The antimicrobial effect was expressed as a diameter of inhibition zone in mm reduced by the inhibition zone of the negative controls, if appropriate.

### 3.7. Statistical Analysis

All data were analyzed using the STATISTICA 10.0 software (Statsoft Inc.). Experimental results were presented in tables as the mean ± standard deviation of three independent replications. Data were subjected to variance analysis (ANOVA) and the Newman–Keuls post hoc test was carried out to identify significant differences between the extract types. Mean values with *p* < 0.05 were considered statistically significant. Pearson correlations were performed to observe the possible correlation between the phenolic profile, antioxidant capacity, and detected antimicrobial activity.

## 4. Conclusions

The phytochemical analysis of the aerial parts of two yarrow species, *Achillea lingulata* and the endemic *Achillea abratonoides*, was performed for the first time. The phenolic composition of four extracts with different polarities suggested that yields of chloroform extracts were the highest, and that they had the biggest diversity of phenolic compounds detected. In addition, ethanolic extracts revealed the strongest antioxidant activities and the ability to suppress the growth of selected microorganisms. Presented results suggest that both investigated *Achillea* species have strong potential for use in different pharmaceutical preparations.

## Figures and Tables

**Table 1:** Yields (%) of obtained extracts of two *Achillea* species.

Species	Plant Organ	Petrol Ether	Chloroform	Ethanol	Water
*A. lingulata*	inflorescence	2.62	11.50	2.00	6.20
	vegetative part	2.86	18.80	3.00	6.30
*A. abrotanoides*	inflorescence	2.92	18.50	3.10	6.60
	vegetative part	2.74	11.00	2.40	5.70

**Table 2:** Phenolic composition (µmol/g) of the extracts of the two *Achillea* species.

Compound	Plant Part	*Achillea lingulata*				*Achillea abrotanoides*			
		PE ^1^	CH ^2^	ET ^3^	W ^4^	PE	CH	ET	W
*p*HBA **^8^**	INF ^5^	21.467 ^a^ ± 4.032	53.927 ^a^ ± 3.366	13.805 ^c^ ± 2.782	nd ^7^	25.150 ^a^ ± 3.008	205.073 ^b^ ± 8.626	24.831 ^d^ ± 0.217	nd
	VEG ^6^	nd	nd	49.327 ^b^ ± 0.986	nd	21.763 ^b^ ± 1.561	28.959 ^e^ ± 3.987	27.691 ^d^ ± 2.765	53.015 ^a^ ± 11.547
*m*HBA ^9^	INF	nd	nd	1.308 ^e^ ± 0.274	1.108^g^ ± 0.069	nd	nd	nd	3.106 ^de^ ± 0.038
	VEG	nd	nd	1.277 ^e^ ± 0.208	nd	nd	nd	nd	nd
23DHBA ^10^	INF	nd	nd	nd	2.196 ^g^ ± 0.203	nd	nd	nd	0.550 ^e^ ± 0.032
	VEG	nd	nd	nd	nd	nd	6.976 ^gh^ ± 0.495	nd	nd
SA ^11^	INF	13.639 ^b^ ± 0.145	33.299 ^b^ ± 2.731	6.892 ^cd^ ± 0.774	nd	16.580 ^c^ ± 0.094	58.153 ^c^ ± 1.777	15.362 ^ef^ ± 0.378	22.235 ^c^ ± 2.229
	VEG	nd	52.029 ^a^ ± 8.987	9.627 ^c^ ± 0.431	4.332 ^f^ ± 0.010	22.375 ^ab^ ± 1.462	27.830 ^e^ ± 0.068	4.086 ^g^ ± 0.153	33.827 ^b^ ± 4.044
ChA ^12^	INF	nd	0.909 ^d^ ± 0.056	0.026 ^d^ ± 0.002	6.055 ^e^ ± 0.077	nd	13.772 ^f^ ± 0.484	0.129 ^h^ ± 0.010	2.884 ^de^ ± 0.221
	VEG	0.691 ^b^ ± 0.072	0.600 ^d^ ± 0.008	0.020 ^d^ ± 0.001	4.066 ^f^ ± 0.431	4.224 ^d^ ± 0.290	1.032 ^hi^ ± 0.014	0.051 ^h^ ± 0.002	9.256 ^d^ ± 1.218
CA ^13^	INF	nd	8.624 ^c^ ± 0.120	3.267 ^d^ ± 0.122	12.126 ^c^ ± 0.186	nd	16.049 ^f^ ± 2.010	2.917 ^gh^ ± 0.122	8.730 ^d^ ± 3.587
	VEG	nd	12.908 ^c^ ± 0.264	3.185 ^d^ ± 0.285	9.182 ^d^ ± 0.462	nd	8.653 ^gh^ ± 0.042	2.551 ^gh^ ± 0.009	11.276 ^d^ ± 0.776
*p*CA ^14^	INF	nd	nd	nd	nd	nd	40.655 ^d^ ± 8.395	19.837 ^e^ ± 0.715	37.786 ^b^ ± 7.975
	VEG	nd	nd	nd	nd	nd	nd	14.340 ^f^ ± 0.492	nd
FA ^15^	INF	nd	nd	nd	0.848 ^h^ ± 0.004	nd	nd	nd	0.981 ^e^ ± 0.091
	VEG	nd	nd	nd	0.202 ^h^ ± 0.012	nd	nd	24.756 ^d^ ± 1.377	0.219 ^e^ ± 0.025
RA ^16^	INF	nd	nd	nd	54.359 ^a^ ± 0.282	nd	207.473 ^b^ ± 17.557	nd	26.866 ^c^ ± 0.513
	VEG	nd	nd	nd	nd	nd	nd	nd	nd
Quercitrin	INF	nd	nd	nd	nd	nd	nd	33.685 ^c^ ± 3.462	31.219 ^b^ ± 0.241
	VEG	nd	nd	nd	nd	nd	0.590 ^i^ ± 0.033	nd	nd
Naringin	INF	nd	nd	nd	nd	nd	nd	35.326 ^c^ ± 2.285	nd
	VEG	nd	nd	nd	nd	nd	0.727 ^i^ ± 0.052	nd	nd
Catechin	INF	nd	nd	nd	nd	nd	nd	nd	nd
	VEG	nd	nd	nd	nd	nd	0.894 ^i^ ± 0.047	nd	nd
Morin	INF	nd	nd	1.718^d^ ± 0.321	nd	nd	nd	23.889^d^ ± 2.895	nd
	VEG	nd	nd	2.860 ^d^ ± 0.002	nd	nd	1.175 ^hi^ ± 0.015	23.166 ^d^ ± 0.903	nd
Apigenin	INF	nd	38.268 ^b^ ± 3.845	2.785 ^d^ ± 0.646	nd	nd	11.495 ^fg^ ± 0.170	0.637 ^h^ ± 0.076	31.647 ^b^ ± 0.913
	VEG	nd	nd	165.688 ^a^ ± 9.680	nd	nd	1.249 ^hi^ ± 0.027	112.010 ^a^ ± 6.564	nd
Naringenin	INF	nd	nd	nd	nd	nd	362.662 ^a^ ± 4.922	95.814 ^b^ ± 7.992	nd
	VEG	nd	nd	nd	nd	nd	nd	nd	nd
Kaempherol	INF	nd	nd	nd	nd	nd	nd	nd	nd
	VEG	nd	nd	nd	nd	nd	0.917 ^i^ ± 0.030	nd	nd
Chrysin	INF	nd	nd	nd	nd	nd	nd	nd	nd
	VEG	nd	nd	nd	nd	nd	3.314 ^hi^ ± 0.186	nd	nd
Pinocembrin	INF	nd	nd	nd	nd	nd	nd	nd	nd
	VEG	nd	nd	nd	nd	nd	1.510 ^hi^ ± 0.065	nd	nd
Galangin	INF	nd	nd	nd	nd	nd	nd	nd	nd
	VEG	nd	nd	nd	nd	nd	1.048 ^hi^ ± 0.189	nd	nd
Hesperetin	INF	nd	9.895 ^c^ ± 1.297	0.253 ^d^ ± 0.060	nd	nd	nd	nd	nd
	VEG	1.408 ^b^ ±0.118	1.284 ^d^ ± 0.021	0.080 ^d^ ± 0.002	27.549 ^b^ ± 1.769	nd	nd	nd	nd
Rutin	INF	nd	11.910 ^c^ ± 1.883	0.294 ^d^ ± 0.011	nd	nd	nd	nd	nd
	VEG	nd	1.128 ^d^ ± 0.032	0.221 ^d^ ± 0.001	nd	nd	nd	nd	nd

^1^ Petrol ether, ^2^ chloroform, ^3^ ethanol, ^4^ water, ^5^ inflorescences, ^6^ vegetative part, ^7^ not detected, ^8^ *p*-hydroxybenzoic acid, ^9^ *m*-hydroybenzoic acid, ^10^ 2,3-dihydroxybenzoic acid, ^11^ salicylic acid, ^12^ chlorogenic acid, ^13^ caffeic acid, ^14^ *p*-coumaric acid, ^15^ ferulic acid, ^16^ rosmarinic acid. The values within one row followed by the same letter do not differ significantly after factorial ANOVA post hoc Newman–Keuls analysis at a significance level of *p* < 0.01.

**Table 3:** Antimicrobial potential of the extracts of two *Achillea* species and selected phenolic compounds.

Species	Extract	Plant Part	*Salmonella* *Abony*	*Escherichia* *Coli*	*Enterococcus* *Faecalis*	*Staphylococcus* *Aureus*	*Candida* *Albicans*
*Achillea* *lingulata*	PE ^1^	INF ^4^	12.00 ± 1.00	15.33 ± 0.58	13.00 ± 0.00	11.67 ± 0.58	18.33 ± 2.52 *
		VEG ^5^	nd ^6^	12.00 ± 0.00	13.00 ± 0.00	12.33 ± 0.58	20.00 ± 2.00 *
	CH ^2^	INF	nd	13.00 ± 0.00 *	13.33 ± 1.53	13.33 ± 0.58	20.00 ± 2.00 *
		VEG	nd	15.67 ± 1.15 *	13.67 ± 0.58	15.00 ± 0.00	19.67 ± 0.58 *
	ET ^3^	INF	15.33 ± 0.58 *	13.00 ± 0.00 *	16.67 ± 2.08	16.00 ± 0.00	25.33 ± 2.51 **
		VEG	12.33 ± 0.58	13.00 ± 1.73 *	14.67 ± 0.58	12.67 ± 0.58	16.33 ± 0.58 *
*Achillea* *abrotanoides*	PE	INF	13.00 ± 0.00	14.33 ± 1.15 *	nd	nd	20.00 ± 0.00 *
		VEG	nd	nd	12.33 ± 0.58	13.33 ± 1.53	18.00 ± 1.73 *
	CH	INF	nd	12.67 ± 1.15 *	14.33 ± 1.15	13.33 ± 0.58	40.00 ± 0.00 **
		VEG	nd	14.00 ± 1.73 *	14.00 ± 1.00	17.67 ± 0.58	nd
	ET	INF	13.67 ± 0.58	14.00 ± 1.00 *	26.00 ± 0.00 **	14.33 ± 0.58	21.67 ± 2.89 *
		VEG	14.33 ± 1.15 *	15.33 ± 1.15 *	18.00 ± 1.00 *	15.67 ± 0.58	24.00 ± 2.00 **
Antibiotic/antimycotic			17.00 ± 1.00	14.33 ± 2.08	19.33 ± 0.58	34.33 ± 2.08	19.67 ± 1.15
Phenolic compound	*p*-Hydroxybenzoic acid		12.33 ± 1.53	12.00 ± 0.00	12.67 ± 1.15	nd	18.67 ± 2.89 *
	Salicylic acid		13.50 ± 0.71	13.00 ± 1.73	13.00 ± 1.73	nd	25.33 ± 0.58 **
	Chlorogenic acid		nd	nd	nd	nd	14.33 ± 0.58
	Caffeic acid		13.00 ± 1.41	15.00 ± 1.00 *	12.00 ± 0.00	nd	nd
	*p*-Coumaric acid		13.67 ± 0.58	11.33 ± 0.58	13.00 ± 0.00	nd	17.00 ± 0.00 *
	Ferulic acid		nd	14.67 ± 0.58 *	nd	nd	nd
	Rosmarinic acid		nd	13.33 ± 1.15	12.67 ± 1.15	nd	17.33 ± 1.15 *
	Quercetin		nd	nd	15.67 ± 1.53	13.67 ± 0.58	18.00 ± 1.73 *
	Naringenin		nd	12.33 ± 0.58	23.00 ± 1.00 **	27.33 ± 2.31 *	22.33 ± 2.52 **
	Morin		14.33 ± 0.58 *	11.33 ± 0.58	18.00 ± 1.73 *	12.00 ± 1.00	19.67 ± 2.52 *

^1^ Petrol ether; ^2^ chloroform; ^3^ ethanol; ^4^ inflorescence; ^5^ vegetative part; ^6^ not detected; * equally as effective as the antibiotic/antimycotic; ** statistically significantly more effective than the antibiotic/antimycotic at significance level *p* < 0.05 after ANOVA post hoc Newman–Keuls analysis.

## Data Availability

The data presented in this study are available on request from the corresponding author.

## Sample Availability

Samples of the extracts are not available, but plant vouchers are deposited at the herbarium of the Department of Biology, Faculty of Science, the University of Sarajevo under the voucher no. LERP 355 and LERP 356.
